# Integrated eco-economic zoning and carbon neutrality zoning into the PLUS model to simulate land use change in the Guangdong–Hong Kong–Macao Greater Bay Area

**DOI:** 10.7717/peerj.20610

**Published:** 2026-01-27

**Authors:** Mingsong Zhan, Yinyin Xu, Yue Yu, Chong Liu, Fan Wang

**Affiliations:** 1 Shenyang University, Shenyang, Liaoning, China; 2 Guangzhou Vocational University of Science and Technology, Guangzhou, Guangdong, China; 3 Northeastern University, Shenyang, Liaoning, China

**Keywords:** Land use simulation, Partition, Ecological risk, Carbon neutrality, PLUS model, Guangdong-Hong Kong-Macao Greater Bay Area

## Abstract

Numerous studies have simulated land use dynamics in megacity regions for sustainable development and urban planning. However, many existing studies apply uniform transition rules across entire regions, ignoring the spatial heterogeneity among subregions. Guangdong–Hong Kong–Macao Greater Bay Area (GBA), one of the three typical urban agglomerations in China, witnesses increased ecological risks and carbon emissions. Aiming to strengthen environmental zoning control, we conducted partition and land use change simulation research. Taking GBA as the study area, we proposed two comprehensive partitions and compared their effects on the simulation accuracy of Patch-generating Land Use Simulation Model (PLUS). One partition was eco-economic zoning, and another was carbon neutrality zoning. The results showed that both partitioned strategies improved accuracy by more than 25% compared to the whole-region simulation. Additionally, the accuracy of the PLUS model varied in sub-partitions. The implementation of PLUS simulations based on different partitions provides a deeper understanding of the spatial differentiation mechanisms of land conversion rules, thereby supporting differentiated zoning control.

## Introduction

Land use system characterizes human-land relationships (Haase et al., 2012; van Asselen et al., 2013). With socioeconomic development, human-induced perturbations became more intensive and might exceed natural variability (Song et al., 2018; Steffen et al., 2011). Consequently, humanity faces a series of global environmental issues, such as biodiversity decline, global warming, and intensified pollution (Foley et al., 2005; Kulmala et al., 2021; Li et al., 2022). These issues can be attributed to the intense human activities, especially the urbanization (Tsiakmakis et al., 2017). Urban areas, as the most active economic regions, cause the ecological risks and produce approximately 70% of the global carbon emissions (Cai & Zhang, 2014). The global urban area has reached 6.5 × 10^5^ km^2^ and supports more than half of the world’s population (Liu et al., 2020). On the one hand, rapid urban expansion occupies natural land (*i.e.,* forestland, wetland), and on the other hand, urban aggregation releases considerable pollution. Both processes ultimately lead to increased ecological risks and a decline in ecosystem services (Seto, Gueneralp & Hutyra, 2012; Wang et al., 2019; Zhou et al., 2022), which in turn impact sustainable urban development and human well-being (Grimm et al., 2008; Wu, 2014). At present, urbanization and its eco-environmental effects have become an important research topic worldwide (Lahiani et al., 2021; Ouyang et al., 2016; Pereira & Baro, 2022; Pickett et al., 2016; Shah et al., 2023; Tao et al., 2021).

High-precision land use simulation is vital to urban planning. Spatial heterogeneity is a key bottleneck affecting simulation accuracy. Partition and spatially partitioned transition rules are effectively used to solve the issue of spatial heterogeneity. There are two main methods for partitioning: administrative district partitioning method and the spatial clustering partition method (Lu, Laffan & Pettit, 2022). Lu, Laffan & Pettit (2022) found that the simulation result became more accurate and robust under two sub-regions simulations. It has been found that spatially partitioned transition rules generate more accurate and stable results than calibrated rules applied to the entire study area, with the mean producer’s spatial accuracy increasing by 72.07% and 75.59% in the two sub-regions. Similarly, Xu et al. (2021) proposed a dual-constrained cellular automata (CA) model and applied it in the Dianchi Lake watershed with a significantly improved accuracy. Specifically, the Kappa index of the partitioned simulation results has increased by 10% compared to the non-partitioned CA model simulation results. Some research findings indicated that the partitioned urban CA models significantly improved the overall accuracy (Ke, Qi & Zeng, 2016; Qian et al., 2020; Wang et al., 2023; Xia & Zhang, 2021). For instance, in one partition study based on the evaluation of land development potential, the Guangdong–Hong Kong–Macao Greater Bay Area (GBA) was divided into five sub-regions, and ultimately the accuracy of the partitioned urban CA models was improved by 7.2% (Wang et al., 2023). Transition rules are perhaps the most significant component in the CA models. Therefore, it is insufficient to adopt the same transition rules across large areas due to the high heterogeneity. Currently, the impact of different partitions on land use simulation accuracy in urban agglomerations remains little understood.

The GBA is the most developed region in China (Zhang et al., 2022). Along with the release of many favorable policies and plans, the GBA tends to face great opportunities to achieve high-quality economic development (Li et al., 2019). Rapid urban expansion has resulted in the significant increase in ecological risk and carbon emissions (Peng et al., 2015). At present, there are large differences in ecological risk, economic development levels, and carbon emissions. For instance, ecological risk exhibits a spatial distribution pattern of being high in the central area and low in the surrounding areas, showing a certain degree of spatial agglomeration (Jiang et al., 2021). The GDP of the east bank of the Pearl River accounts for 77% of the whole region, while the west bank only accounts for 23%. As is well known, GDP is one of the most important socio-economic factors, which could directly reflect the level of economic development. GDP is closely related to carbon emissions. Therefore, there are significant differences in the spatial structure and intensity of land use changes at different economic levels. Thus, it is meaningful to take the GBA as a case to conduct partition research for divisional strategy. It can also provide implications for other similar regions. Aimed at the key ecological issues, two partitions were integrated: one partition was eco-economic zoning, and another was carbon neutrality zoning. Compared to ecological zoning and ecological functional zoning, eco-economic zoning emphasize the possibility and severity of damage to ecosystems and their functions in the event of interference, carbon neutrality zoning not only focuses on the carbon sink function of ecosystems, but also pays more attention to the carbon emission function of cities. In order to reduce regional ecological risks, the conversion of forests, wetlands, and grasslands in extremely high-risk areas into construction or cultivated land will be prohibited; land use conversion in high-risk areas can be allowed, but strict control measures are in place; land use conversion in low-risk areas is shifting towards reducing ecological risks. High carbon emission zones, industrial green transformation or relocation; medium carbon emission zone, industrial upgrading or other green industry substitution; low carbon emission zones strictly limit the introduction of high carbon emission industries. The main innovation of this study was two partition strategies were proposed and applied to land use partition simulation in considering the complex geographical conditions and economic development levels region.

The land use model (LUM) help to explore and determine the underlying driving factors of land use changes. The common LUM include the cellular automata-based model and the Conversion of Land Use and its Effects at Small regional extent (CLUE-S) (Bunyangha et al., 2021; Liu et al., 2014; Verburg et al., 2002). The Patch-generating Land Use Simulation Model (PLUS) have two superiorities, one is employed a random forest classification algorithm to capture the non-linear relationships among multiple driving factors; another is a multi-type random patch seeding mechanism, which effectively simulate the competitive relationship between various types of land use (Liang et al., 2021). Currently, PLUS has been widely used in different regions at multi scales. Compared to other LUMs, the PLUS model have a higher accuracy when simulated future city land use change (Kou et al., 2023; Yu et al., 2023). Thus, we adopted PLUS model and comprehensive partitions to achieve a higher simulation accuracy. The main contributions of this study are as follows: (1) The spatial–temporal characteristics of land use change in the GBA from 2010 to 2020 were identified; (2) two comprehensive partitions were proposed; (3) the differences in the accuracy of the partitioned simulation results were tested. These findings can enrich land use zoning simulation, promote precise assessment of ecological security and carbon neutrality, and support urban sustainable development.

## Materials & Methods

**Figure 1:**
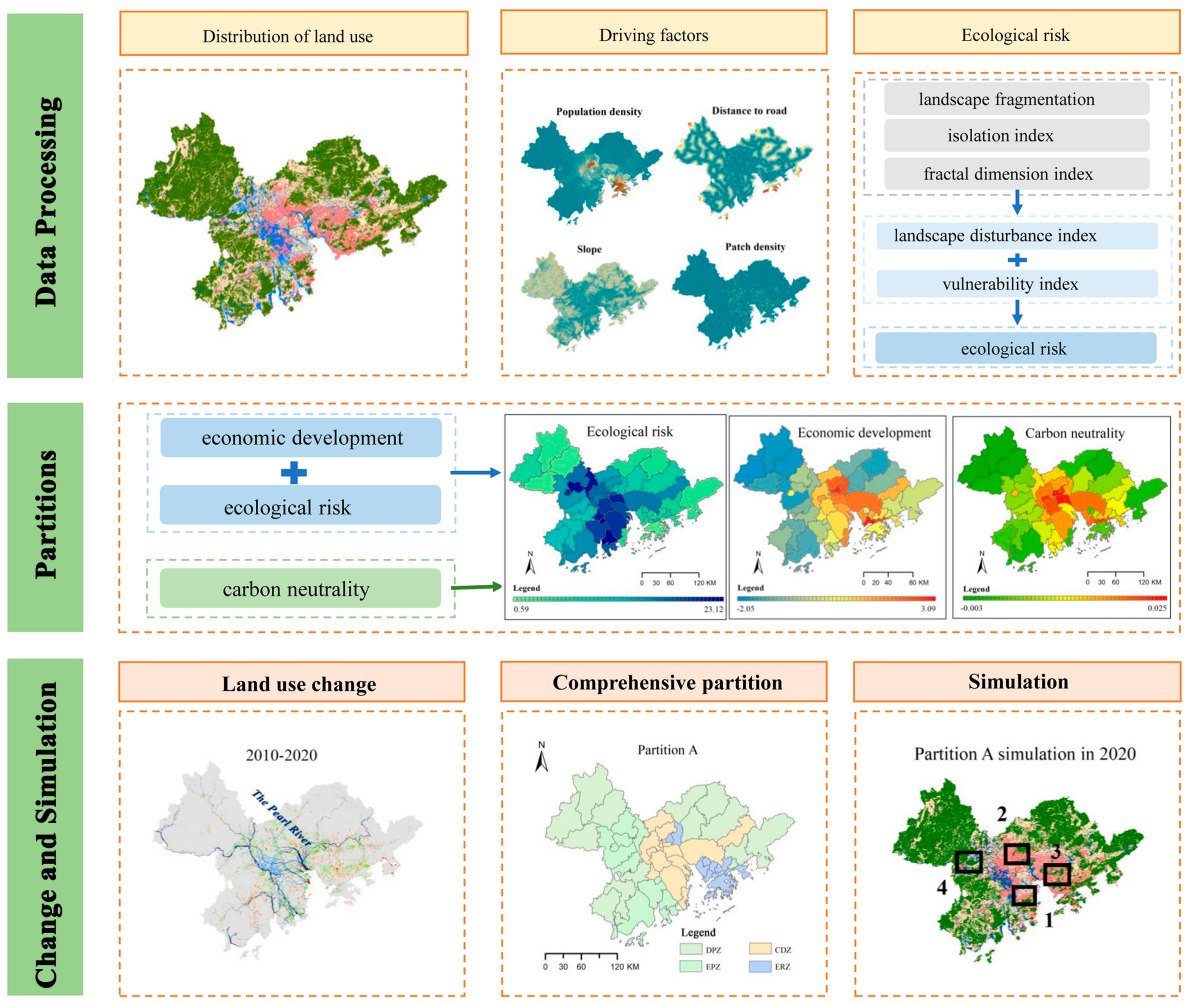
Research steps of this study.

In this study, two comprehensive partitions were first mapped. Then, the PLUS models were applied to make simulations: one was based on the entire region, and the other two were based on different comprehensive partitions. Finally, the results of the three PLUS models were compared to assess the simulation accuracy (Fig. 1).

### Study area

**Figure 2:**
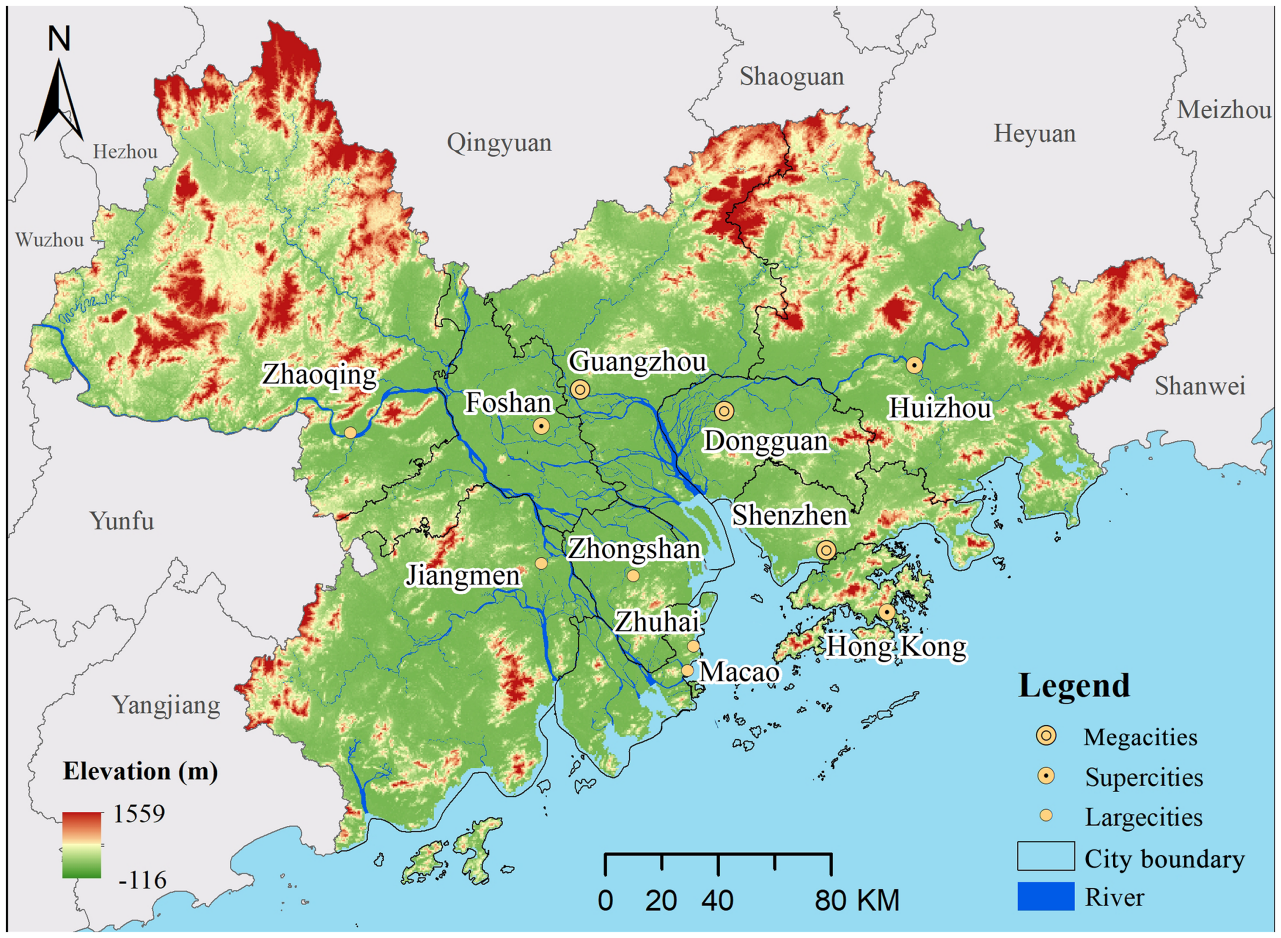
Location of the study area.

The GBA (111°20′–115°24′E, 21°32′–24°26′N) lies in southern China with an area of nearly 56,000 km^2^ (Fig. 2). Its terrain is high in the northwest and low in the southeast with altitudes of -165–1,559 m. The central part is plain, its surrounding is mountains and hills. The whole region is dominated by tropical and subtropical monsoon climate, with mean annual temperature of 21.8 °C and annual rainfall of 1,789.3 mm. The GBA comprises of nine inland cities and two special administrative regions. According to ‘Notice of the State Council on Adjusting the Standards for Urban Scale Classification (2014)’, cities with a permanent population of over 10 million in urban areas are considered as megacities; Cities with a permanent population of five million to 10 million in urban areas are considered as supercities; Cities with a permanent population of one million to five million in urban areas are considered as large cities. Therefore, the GBA contains eleven cities, including three megacities with a population of more than 10 million, namely Guangzhou, Shenzhen and Dongguan; three supercities with a population of five to 10 million, namely Hong Kong, Foshan and Huizhou; and five large cities with less than five million, namely Macao, Jiangmen, Zhongshan, Zhaoqing and Zhuhai.

With population growth and rising consumption levels, land use has changed significantly, accompanied by substantial farmland loss and urban sprawl (Zhang et al., 2022). As a result, the ecological risk is constantly increasing over time (Lu, Laffan & Pettit, 2022). In terms of carbon emissions, the amount increased by 184 Mt from 2000 to 2016, with a growth rate of 7% over 2000–2011 (Zhou et al., 2018).

### Data sources

Land use data for 2010, 2015, and 2020 (spatial resolution: 30 m), which adopt a secondary classification system, were obtained from the Chinese Academy of Sciences (http://www.resdc.cn/) (Table 1) (Liu et al., 2010). We applied first class type, namely farmland, wetland, grassland, forestland, and construction land. The population density, GDP, elevation, and roads were collected from the Resources and Environmental Scientific Data Center (RESDC) (http://www.resdc.cn/). High-speed railways were obtained from the Baidu Map (https://map.baidu.com). Points of interest (POIs) were derived through network crawling, including restaurants, bus stops, metro stations, manufacturers, schools, hospitals, and residences. Validating by field points, the accuracy of POIs reached at 90%. County-level CO_2_ emissions and sequestration data were downloaded from previous studies (https://www.ceads.net.cn/) (Chen et al., 2020). Those data were resampled to 500 m and unified coordinates to the Albers coordinate system.

**Table 1:** Description of data used in this study.

**Data**	**Data source**
Land use data	The Chinese Academy of Sciences (http://www.resdc.cn/)
Population density	The Resources and Environmental Scientific Data Center (http://www.resdc.cn/)
Elevation	The Resources and Environmental Scientific Data Center (http://www.resdc.cn/)
GDP	The Resources and Environmental Scientific Data Center (http://www.resdc.cn/)
Different levels of roads	The National Earth System Science Data Center (http://wdcrre.data.ac.cn/)
Railways/High-speed railways	The Baidu Map (https://map.baidu.com)
POIs	Network crawling
Ecological protection redlines	The Department of Ecology and Environment of Guangdong Province (http://gdee.gd.gov.cn/)

### Comprehensive partitions

Previous studies have verified the feasibility of the comprehensive partitions in other regions (Sun et al., 2020; Xu et al., 2022). Two comprehensive partitions were adopted, one partition was eco-economic zoning, and another was carbon neutrality zoning. In the view of governance philosophy, the core concept of two comprehensive partitions is to achieve harmonious coexistence between humans and nature, the former focuses on the coordinated development of ecological and economic systems, while the latter adjusts land use structure from the perspective of carbon neutrality. In the perspective of governance measures, the former focuses on both ecosystem protection and restoration as well as economic development, while the latter emphasizes emission reduction in high carbon emission areas. The indicators for partitioning were closely related to land use types, indicating that the comprehensive partitions have potential values, and another was carbon neutrality zoning

#### Eco-economic zoning

Following Sun et al. (2020), the region can be divided into four categories based on ecological risk and economic development, namely coordinated development zone (CDZ), economic poverty zone (EPZ), ecological risk zone (ERZ), and dual pressure zone (DPZ) (Table 2) (Wu et al., 2018).

**Table 2:** Characteristics of partitions base on ecological risk and economic development.

**Category**	**Division standard**	**Regional characteristics**
Coordinated development zone	ERI>its median value and EDI>its median value	Two indicators are high.
Ecological risk zone	ERI<its median value and EDI>its median value	The ecological risk is low; and the economic development is high.
Economic poverty zone	ERI>its median value and EDI<its median value	The ecological is high; the economic development is low.
Dual pressure zone	ERI<its median value and EDI<its median value	Two indicators are low.

**Notes.**

ERI represents ecological risk index, EDI represents economic development index.

The ecological risk index can be expressed as follows:

(1) \begin{eqnarray*}{\mathrm{ESI}}_{\mathrm{k}}=\sum _{\mathrm{i}=1}^{\mathrm{n}} \frac{{\mathrm{A}}_{\mathrm{ki}}}{{\mathrm{A}}_{\mathrm{k}}} (1-10\times {\mathrm{R}}_{\mathrm{i}})\end{eqnarray*}

where, ESI_K_ is the ecological risk index; A_ki_ and A_k_ are the area of the ith land use class in the kth region and kth region, respectively. R_i_ is the loss index of the ith landscape class. The detail calculation process could be seen in Appendix SA.

The economic development index was acquired through principal component analysis (PCA) based on socioeconomic statistical data in 2021. We collected 10 indicators, which including social and economic development aspects. PCA was advantageous in reducing the dimensions of the data and eliminating multicollinearity between features (Nguyen & Ye, 2015). Three principal components (*E*_1_, *E*_2_, *E*_3_) were obtained, with cumulative contributions exceeding 0.84 (Table 3). This suggests that the three principal components can replace the original variables.

**Table 3:** Total variance explained.

Component	Standard deviation	Proportion of variance (%)	Cumulative proportion (%)
GDP	2.26	0.51	0.51
Per capita GDP	1.52	0.23	0.74
Primary industry	1.00	0.10	0.84
Secondary industry	0.74	0.05	0.90
Tertiary industry	0.65	0.04	0.94
Local governments’ general public income	0.55	0.03	0.97
Per capita disposable income of urban residents	0.38	0.01	0.98
Investment in fixed assets	0.33	0.01	0.99
Total retail sales of social consumer goods	0.22	0.01	1.00
Total imports and exports	0.00	0.00	1.00

The economic development index can be expressed as:

(2) \begin{eqnarray*}E={\alpha }_{1}{E}_{1}+{\alpha }_{2}{E}_{2}+{\alpha }_{3}{E}_{3}\end{eqnarray*}

where, *α* is the coefficients, which is determined by the percentage of variance.

#### Carbon neutrality zoning

China announced to achieve carbon neutrality by 2060 to address climate challenges. Carbon neutrality refers to a state where carbon emissions are balanced by carbon sequestration. To achieve carbon neutrality by 2060 for China, the measures must suit local conditions (Hu et al., 2022). Thus, land space zoning can provide support for land use optimization.

Chen et al. (2020) developed a set of long term series CO_2_ emissions data. Compared with existing studies (Guan et al., 2012; Luo et al., 2021; Shan et al., 2018), the simulated CO_2_emissions are reliable. Based on the carbon neutrality data, the natural breakpoint method was used to divide the region into three zones: the low carbon sequestrate (LCS), medium carbon sequestrate (MCS), and high carbon sequestrate (HCS).

### PLUS model

Compared with existing CA-based LUMs, the PLUS model has a higher accuracy through further understanding the relationships between land use types and driving factors by random forest classification algorithm, and could simulate land use change at patch level (Liang et al., 2021). The formulas could be seen in Appendix SB.

#### Driving factors

**Figure 3:**
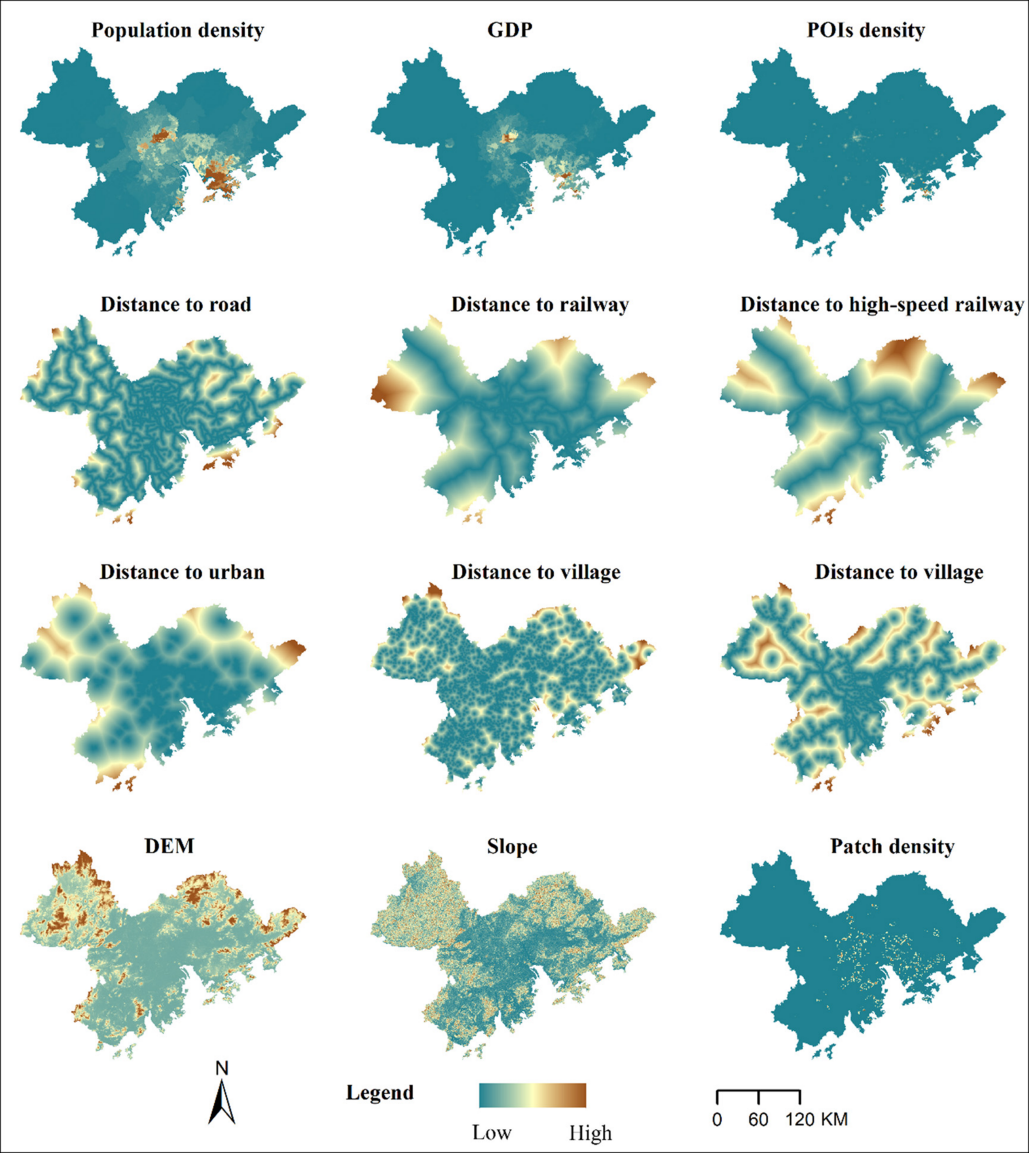
Spatial distribution map of driving factors.

Land use change is closely linked to population, economic, transportation, and natural factors. The sustained growth of urban population and economic development are key force driving the expansion of urban construction land. Transportation and natural factors define urban development trajectories and patterns. To eliminate multicollinearity among factors, we conducted a variance inflation factor (VIF) test. If the VIF value of two factors exceeded 5, we removed one of them. Eventually, a total of 12 driving factors were selected (Zhuang et al., 2022), including socioeconomic factors (GDP, population density, POIs density, distance to transportation line, distance to urban area, distance to villages), topographic factors (elevation, slope), and fragmentation factor (patch density of urban construction land) (Fig. 3). The PLUS model employs the random forest algorithm to individually analyze the driving factors of various types of land use expansion. It obtains the development probability for each land use type, as well as the contribution of driving factors to the expansion of each land use category during the period.

#### Land use simulation

We simulated land use in 2020 based on land use data in 2010. The input land use demand was consistent with actual situations in 2020. Three simulation schemes were conducted, including the entire region simulation and two different partitioned simulations. In eco-economic zoning simulation and carbon neutrality zoning simulation, the region was divided into four and three sub-regions, respectively. During simulation, each sub-region had its own set of operating parameters. For example, land use change transfer rules were based on land use conversion from 2010 to 2020 (Table 4). Thus, each sub-region transfer rules could be calculated based on their land use conversion. 0 represents that conversion is not possible, and 1 represents that conversion is allowed. According to relevant studies (Liang et al., 2021), neighborhood can be determined by the proportion of a certain type of land use change area to the total land use change area (Table 5). In the entire region simulation, the neighborhood weights for farmland, forestland, grassland, wetland, and construction land were 0.232, 0.019, 0.041, 0.055, 0.651, respectively.

**Table 4:** Scenario simulation of partition DPZ transfer rules.

	Farmland	Forestland	Grassland	Wetland	Construction land
Farmland	1	0	1	1	1
Forestland	1	1	1	1	1
Grassland	1	0	1	1	1
Wetland	1	1	1	1	1
Construction land	0	0	0	0	1

**Notes.**

0 represents that conversion is not possible, and 1 represents that conversion is allowed.

**Table 5:** Scenario simulation neighborhood weights.

	Farmland	Forestland	Grassland	Wetland	Construction land
Partition DPZ	0.180	0.314	0.009	0.094	0.396
Partition EPZ	0.383	0.112	0.008	0.265	0.231
Partition CDZ	0.023	0.043	0.042	0.431	0.458
Partition ERZ	0.362	0.082	0.032	0.046	0.476
Partition LCS	0.296	0.193	0.010	0.148	0.346
Partition MCS	0.246	0.007	0.013	0.492	0.239
Partition HCS	0.449	0.001	0.064	0.036	0.449

**Notes.**

CDZ, coordinated development zone; EPZ, economic poverty zone; ERZ, eco-logical risk zone; DPZ, dual pressure zone; LCS, the low carbon sequestrate zone; MCS, medium carbon sequestrate zone; HCS, high carbon sequestrate zone.

##### Accuracy test

The comparative analysis was conducted at the whole region and subregion respectively. When the comparison was made at the whole region, there was a need to mosaic the simulation results of the subregion. When the comparison was made at the subregions, the subregion must be extracted from the simulation result of the whole region.

The overall accuracy (OA) and the figure of merit (FoM) are commonly used to assess the accuracy of land use simulation (Liang et al., 2021; Pontius & Schneider, 2001). Kappa and FoM can be expressed as:

(3) \begin{eqnarray*}OA= \frac{B+E}{(A+B+C+D+E)} \end{eqnarray*}

(4) \begin{eqnarray*}FOM= \frac{B}{(A+B+C+D)} \end{eqnarray*}

where B represents the area where both actual and simulated conversions occur, A represents the area where conversion actually occurred but simulation did not occur. C represents the area where the actual conversion occurred but the simulated conversion result was different from the actual one. D represents there is no actual change, but the area where conversion occurs is simulated. E represents the land that is actually unchanged and simulated as unchanged.

The comparative analysis with partitioning as a reference meant that the non partition simulation results were extracted according to the partition results, and the accuracy was compared with the partition simulation results of the GBA. The comparative analysis with non partition as a reference meant that the non partition simulation results were matched according to the partition results, and the accuracy was compared with that of the non partition simulation results of the GBA.

## Results

### Land use dynamics during 2010–2020

**Figure 4:**
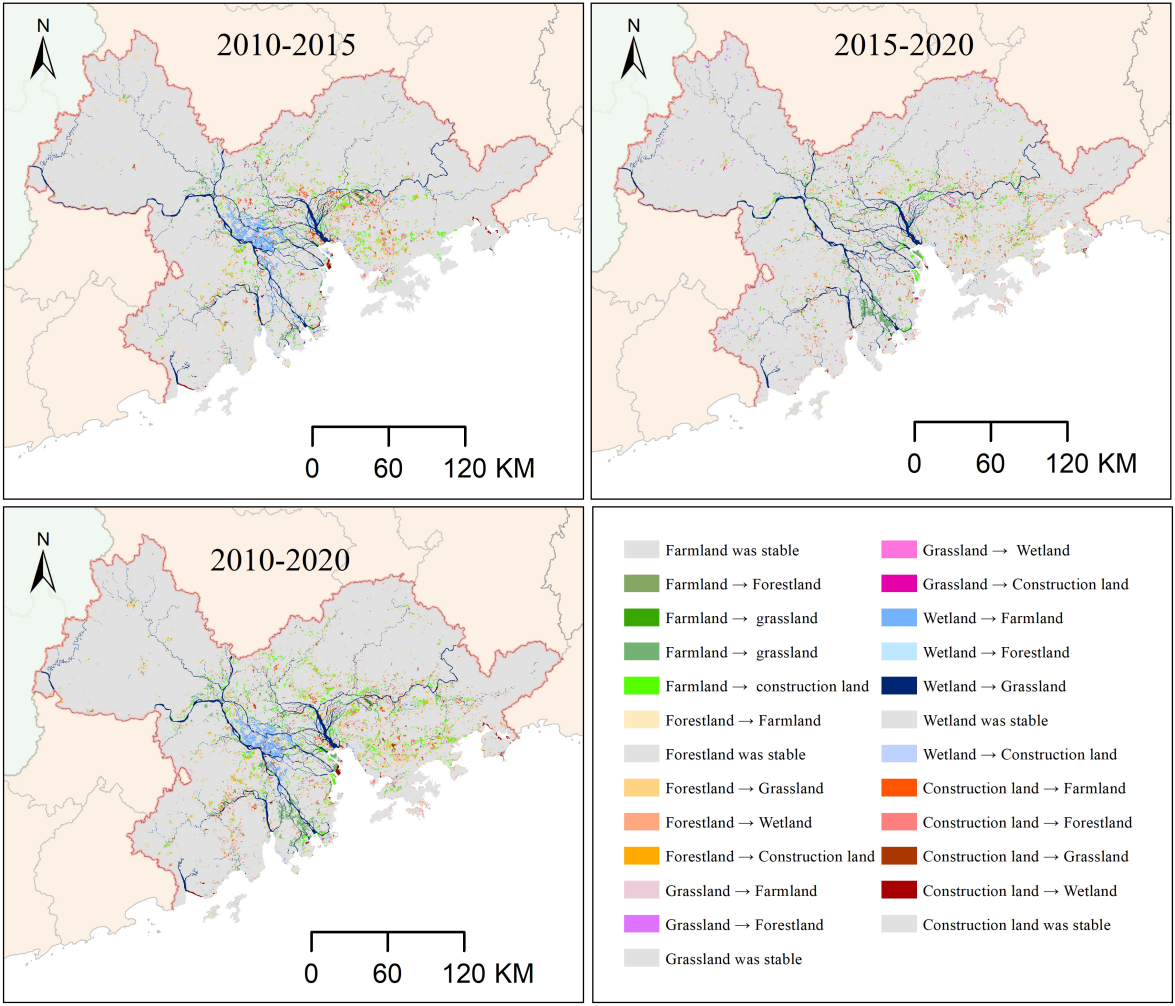
Land use change during 2010–2020.

**Figure 5:**
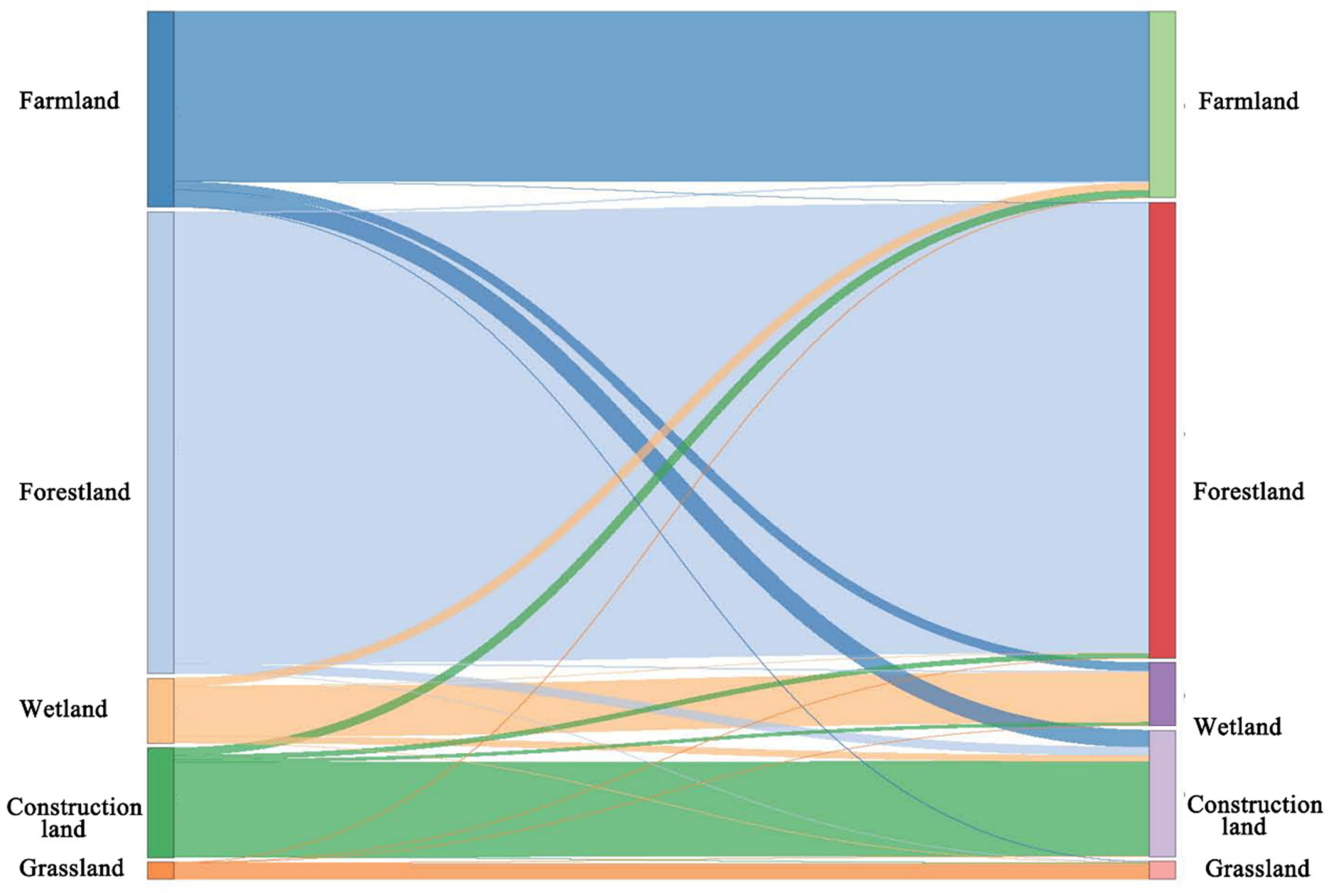
Sankey diagram during 2010–2020.

Farmland and forestland were the dominant land use types during the study period, covering 23.1% and 54.4% of the entire region in 2010, respectively (Fig. 4; Table 6). However, by 2020, farmland and forestland had experienced a continuous decline of 4.7% and 1.3% in area, respectively. Construction land experienced a substantial expansion, with a increase of 11.4% (815.1 km^2^) during 2010–2015 and 5.2% (374.0 km^2^) during 2015–2020. The most widespread land use change in the GBA was the conversion of farmland to construction land, followed by the conversion of forestland to construction land during 2010–2020 (Figs. 4, 5).

**Table 6:** Changes in land use quantity and proportion in the GBA.

**Category**	**Area (km** ^**2**^ **)**	**Proportion (%)**	**Area (km** ^**2**^ **)**	**Proportion (%)**	**Area** **(km** ^**2**^ **)**	**Proportion (%)**
	**2010**	**2010**	**2015**	**2015**	**2020**	**2020**
Farmland	12,690.5	23.1	12,516.8	22.7	12,091.6	21.9
Forestland	29,925.8	54.4	29,586.9	53.6	29,547.5	53.5
Grassland	1,091.6	2.0	1,242.3	2.3	1,169.2	2.1
Wetland	4,202.7	7.6	3,883.9	7.0	4,108.0	7.4
Construction land	7,124.9	12.9	7,940.5	14.4	8,315.0	15.1

### Partition results

**Figure 6:**
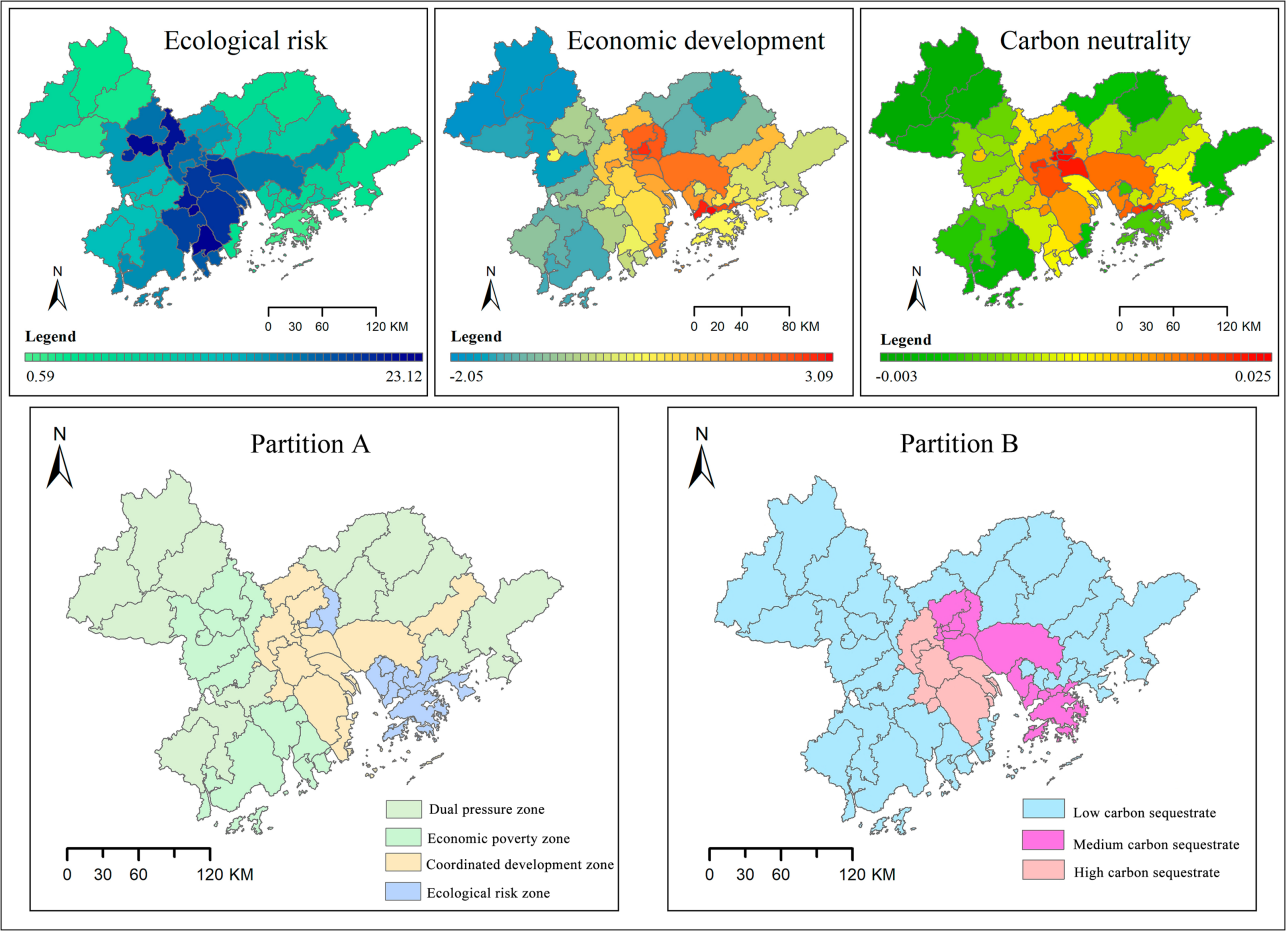
Partition results. Partition A based on ecological risk and economic development index; partition B based on carbon neutrality.

The ecological risk level presented a high trend in the southwest but a low trend in the east bank of Pearl River (Fig. 6). It suggested that land in the southwest was less disturbed. Owing to urbanization, the region in the east bank of Pearl River has suffered severe human disturbances (He et al., 2021). Therefore, the landscape tended to be fragmented, which resulted in the ecological risk index values increasing. The economic development in the central regions was higher than that in the surrounding regions, and the value of GDP of the east bank of Pearl River was higher than that in the west bank of the Pearl River. In terms of the carbon neutrality, the value of the central regions was higher than that in the surrounding regions.

The CDZ was concentrated in Foshan, Zhongshan, Dongguan, and Guangzhou rural areas (Fig. 6). The partition ERZ was mainly situated in Guangzhou core areas, Shenzhen, and Hongkong. The EPZ was concentrated in Jiangmen, Zhaoqing, and Zhuhai urban areas. The DPZ was scattered and mainly distributed in the surrounding regions. The partitioned LCS accounted for most of the area and was mainly distributed in the surrounding area. The partition MCS and partition HCS were concentrated in the central region and separated by the Pearl River (Fig. 6).

**Figure 7:**
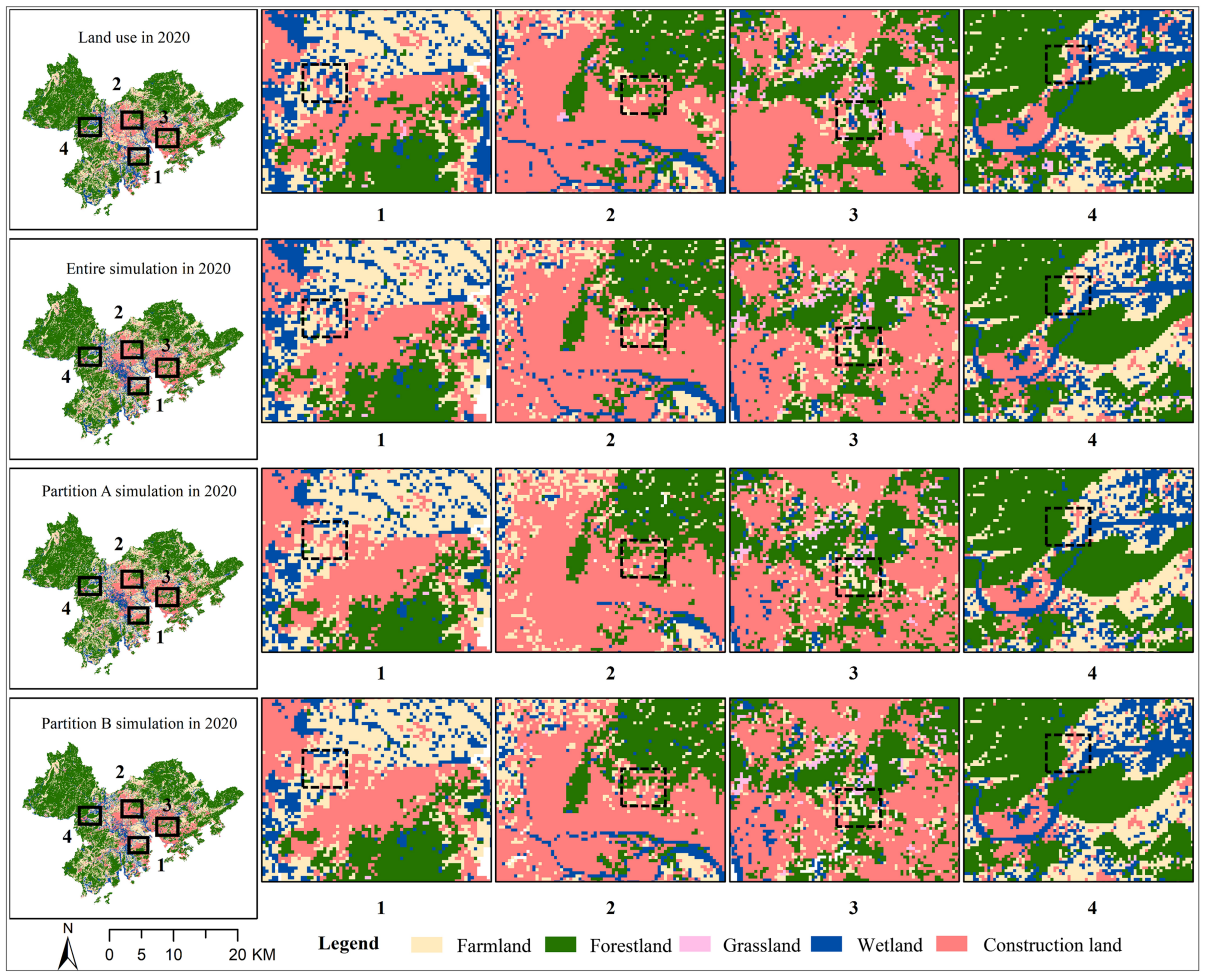
Simulation results of PLUS models with and without considering partitions in 2020.

### Simulation results analysis

The results of entire simulation in 2020, partition A simulation in 2020, and partition B simulation were shown in Fig. 7. The whole-region simulation results showed that the OA was 0.7720 and the FoM was 0.0829. The OA was 0.8454 and the FoM was 0.1146 under eco-economic zoning (Table 7). The OA was 0.7925, and the FoM was 0.1038 under carbon neutrality zoning. This indicated that the accuracy of the PLUS model under the two scenarios improved significantly, with increases of 38.23% for eco-economic zoning and 25.21% for carbon neutrality zoning, compared to the whole-region simulation.

The accuracy of the PLUS model varied with sub partition. The comparative change in OA of each partition was increased. FoM ranged from 0.0728 to 0.1947 under eco-economic zoning. FoM varied from 0.0768 to 0.1710 under carbon neutrality zoning (Table 7). Figure 7 showed the detailed simulated land use patterns of 2020 under different simulation strategies. The whole or partitioned simulation patterns all showed a high spatial consistency with the actual land use pattern.

## Discussion

### Partitions applicability and its effects on land use simulation

The GBA lies in the Pearl River Delta, where is the most advanced agricultural region in China. Large-scale urbanization was inevitable occupied because its lower development costs. With the implementation of farmland requisition-compensation balance and farmland red line, farmland is more effectively protected. Simultaneously, forestland on gentle slopes was converted for urban development. Land use changed induced by urbanization alter regional ecological processes, eventually increased in ecological risk, particularly well-developed areas. Industrial urbanization has not only led to a surge in carbon emissions but also caused a decline in carbon sinks. Ultimately, this goal of carbon neutrality became more difficult achieve.

**Table 7:** Accuracy comparison of each partition simulation results under partitioned scenarios and non partitioned scenarios (extracted each partition) in GBA in 2020.

**Regions**	**Partitioned simulation**		**Whole simulation**	
	**OA**	**FoM**	**OA**	**FoM**
Partition DPZ	0.9057	0.0728	0.8826	0.0585
Partition EPZ	0.8763	0.0844	0.8572	0.0461
Partition CDZ	0.7852	0.1054	0.7578	0.0803
Partition ERZ	0.8545	0.1947	0.7699	0.1683
Partition LCS	0.8292	0.1710	0.7516	0.1233
Partition MCS	0.7885	0.1147	0.7194	0.0982
Partition HCS	0.8097	0.0768	0.7956	0.0531

As the urban expansion slowed down, natural land has been protected to a certain extent (Zhang et al., 2022). Previous researches have studied the ecological security patterns across the GBA by identifying the key ecosystem services (Bi, Xie & Yao, 2020; Gao et al., 2022; Jiang et al., 2021; Wang et al., 2021; Zhang et al., 2023), and our results were consistent with these findings. The economic data have been validated by statistical data, with a high accuracy. Therefore, eco-economic zoning was reliable. In terms of the pattern of carbon neutrality, the results were similar to those from other studies (Li et al., 2023; Lin & Li, 2020; Luo et al., 2021). It suggests that the foundation data for partition based on carbon neutrality was applicable. Ecological risk, economic development and carbon neutrality are strongly related to land use, which guarantees that space zoning based on these indicators can be feasible. Additionally, the two partitions are realistic and have a practical significance.

Theoretically, reasonable space partitioning can improve the performance of land use models. The land use conversion rules of the GBA vary between the whole region and the sub regions, both with a strong spatial heterogeneity. Under partition A, the main reason was that the land use dynamics still varied greatly in sub partitions. Additionally, partitioning increased the complexity and uncertainty of the model. Therefore, eco-economic zoning need to be further studied in other regions. Under carbon neutrality zoning, the ecological resources were mainly distributed in the LCS partition with fewer human activities; thus, the land use changed slowly. The differences of industrial structures in MCS and HCS cause the changes of land use in different ways. For example, the leading industry of Shenzhen is the service industry, which commonly requires less land. In contrast, the leading industry of Foshan is manufacturing, which requires more land use. This indicates that carbon emissions are closely related to land use types, and carbon zoning is more suitable for the partitioned simulation of land use change. In our study, we considered the spatial heterogeneity in view of ecological risk, economic development, and carbon neutrality. Thus, both the accuracy of eco-economic zoning simulation and carbon neutrality zoning simulation improved significantly.

### Zoning method

Spatial heterogeneity exists in land use simulation. The dominant driving factors and their strength vary in different sub partitions. Partitioning the region can be based on land use attributes, administration, and main function (Yin et al., 2018). At present, partitioning the region faces some uncertainties, such as how to determine the thresholds and the number of sub partitions (Xia et al., 2019). A series of classification methods can be used to generate sub partitions, including k-means cluster algorithms, dual-constrained spatial clustering, and self-organizing map (Ke, Qi & Zeng, 2016; Qian et al., 2020; Xia & Zhang, 2021; Xu et al., 2021). But the k-means cluster algorithm has a strong subjectivity, and the number of partitions needs to be determined in advance. Additionally, it treats each pixel as discrete, which neglects spatial autocorrelation. In contrast, self-organizing maps preserve the properties of the whole region well when considering the constraint of neighborhood distance. Therefore, more methods need to test, such as Geodetector, Geo-self-organizing map, and decision trees. Transition rules are also important for the accuracy of the simulation. Previous studies have adopted logistic regression, artificial neural network, and random forest to capture the transition rules (Liao et al., 2016). More methods need to be applied and compared, such as deep learning methods (He et al., 2018).

### Limitations and future research

Although two partitioning strategies have been applied to land use simulation, there are still some limitations that need to be addressed. Firstly, driving factors are stable in the PLUS model, which decreases the reliability of the long term simulation based on the model (Feng et al., 2019). Additionally, the main driving factors vary in different zones. However, we used the same driving factors in different sub-partitions. In future, the main driving factors should be screened out for model simulation. Secondly, the land use data had a coarse spatial resolution, which possibly misses the information at finer resolution. This limits the generalizability of the conclusion. Thirdly, there is a need to put more efforts to investigate the meaning of eco-economic zoning, such as the threshold method and number of the sub-region. Nevertheless, we proposed two zoning strategies separately based on ecological risk and carbon neutrality at urban agglomerations levels and integrated them into partitioned land use simulation, which has a great significance for “current-future” ecosystem management to achieve the balance of economic development and ecological protection. The practical applications contain territorial spatial planning, environmental zoning control, regional coordinated development strategy.

## Conclusions

The dominant landscape types in the GBA were forestland and farmland between 2010 and 2020. The construction land continuously increased (1189.1 km^2^), but the growing rate was slow. Two comprehensive partition categories based on ecological risk, economic development index and carbon neutrality were proposed. By integrating several driving factors (such as socioeconomic factors, natural factors, and fragmentation factors), the PLUS model was adopted to simulate land use change from 2010 to 2020 in GBA. The accuracy of the PLUS model under two scenarios improved significantly, with 38.23% under eco-economic zoning and 25.21% under carbon neutrality zoning when compared to the entire simulation, respectively. Additionally, the accuracy of the PLUS model varied in sub-partitions. Rationality of partitioning in land use simulation should be considered. The implementation of PLUS simulation based on different partitions provides a further understanding of the spatial heterogeneity in land use change. This findings of this study can be generalized to other urban agglomerations. It provides implications for territorial spatial planning, carbon neutrality management, and sustainable development.

## Supplemental Information

10.7717/peerj.20610/supp-1 Supplemental Information 1 Land use transfer matrix (2010–2015, 2015–2020, 2010–2020)

## Additional Information and Declarations

### Data Availability

The following information was supplied regarding data availability: The raw measurements are available in the Supplementary File.
