# Supplementary material for: Integrated eco-economic zoning and carbon neutrality zoning into the PLUS model to simulate land use change in the Guangdong–Hong Kong–Macao Greater Bay Area
Source: PeerJ. 2026 Jan 27;14:e20610. doi: 10.7717/peerj.20610 (PMC12857558; doi:10.7717/peerj.20610)
Supplement: Supplemental Information 1 [file peerj-14-20610-s001.zip › Supplemental Files/Appendix A.docx]

**Appendix A.**

| The formula of $R_{i}$ is:  $R_{i}=D_{i}\times V_{i}$ | (1) |
| --- | --- |

Where, $D_{i}$ and $V_{i}$ are the landscape disturbance index and vulnerability index of the $i$th landscape class, respectively. The formula of $D_{i}$ is:

| $D_{i}=aC_{i}+bN_{i}+cF_{i}$ | (2) |
| --- | --- |

Where, $C_{i}$, $N_{i}$, and $F_{i}$ are the landscape fragmentation index, isolation index, and fractal dimension index of the $i$th class, respectively. Based on previous research, the values of a, b, and c were 0.5, 0.3, and 0.2, respectively.

The landscape fragmentation index measures the number of patches within an area and represents the fragmentation of the landscape. The landscape isolation index displays the degree of separation of patches. The landscape fractal dimension index shows the complexity of patch shape. All landscape indexes were calculated based on Fragstats 4.2.

The landscape vulnerability index measures the resistance of landscape types to external disturbances. The larger the landscape vulnerability index is, the weaker the resistance to external disturbances. According to previous studies, The values of landscape vulnerability of the farmland, forestland, grassland, wetland, and construction land are 4, 2, 3, 5, and 1, respectively. Each indicator was standardized using the MIN-MAX standardization method.
