# Supplementary material for: Integrated eco-economic zoning and carbon neutrality zoning into the PLUS model to simulate land use change in the Guangdong–Hong Kong–Macao Greater Bay Area
Source: PeerJ. 2026 Jan 27;14:e20610. doi: 10.7717/peerj.20610 (PMC12857558; doi:10.7717/peerj.20610)
Supplement: Supplemental Information 1 [file peerj-14-20610-s001.zip › Supplemental Files/Appendix B.docx]

**Appendix B.**

The formulas are as follows:

| $P_{i,k}^{d}(x)=\frac{\sum_{n=1}^{M} I(h_{n}\left( x \right)=d)}{M}$ | (3) |
| --- | --- |
| ${OP}_{i,k}^{d=1,t}=\left\{ \begin{aligned} P_{i,k}^{d}\times\left( r\times\mu_{k} \right)\times D_{k}^{t} if \Omega_{i,k}^{t}=0 and r<P_{i,k}^{d=1} \\ P_{i,k}^{d}\times\Omega_{i,k}^{t}\times D_{k}^{t} all others \end{aligned} \right.$ | (4) |

Where, $P_{i,k}^{d}$ and ${OP}_{i,k}^{d=1,t}$ are the final growth probability of land-use $k$k in unit $i$ and the growth probability surface of the land use, respectively. $I$ is the function of decision sets. $h_{n}\left( x \right)$ donates the predicted land use type of the $n$th decision tree for vector $x$. $M$ represents the total decisions. $r$ is a random value between 0 and 1. $\mu_{k}$ is a user-determined threshold for generating new land-use patches.
